# Atmospheric profiles associated with pyrocumulonimbus in southeast Australia

**DOI:** 10.1038/s41598-025-22530-0

**Published:** 2025-11-04

**Authors:** Caleb S. Wilson, Jason J. Sharples, Jason P. Evans

**Affiliations:** 1 School of Science, University of New South Wales, Canberra, ACT Australia; 2 New South Wales Bushfire and Natural Hazards Research Centre, Sydney, NSW Australia; 3 ARC Centre of Excellence for Climate Extremes, UNSW, Canberra, ACT Australia; 4 Climate Change Research Centre, UNSW, Sydney, NSW Australia; 5 ARC Centre of Excellence for Climate Extremes, UNSW, Sydney, NSW Australia

**Keywords:** Natural hazards, Atmospheric science

## Abstract

Pyrocumulonimbus (pyroCb) are thunderstorms generated by wildfires. They present significant risks to first responders and civilian populations in southeast Australia and in many regions around the world. PyroCb are increasingly common in southeast Australia, with the majority of recorded events occurring in the last 20 years. We constructed, examined, and statistically compared median atmospheric profiles for pyroCb-producing and large standard (non-pyroCb-producing) wildfires in the southeast Australian mainland over a 30-year time period (1991–2020). We found that pyroCb in southeast Australia frequently develop on days with hot, relatively dry, very unstable, and moderately windy surface and low-level conditions that are favourable for wildfire spread. These conditions often occur in conjunction with steep mid-level lapse rates and significant diurnal mid-level moisture advection, resulting in conditions at least conditionally favourable for high-based thunderstorm development. PyroCb and standard wildfire events have similar wind profiles, particularly in the lower-levels, but pyroCb tend to be associated with lower winds in the mid- and upper-levels of the troposphere. However, variability within the pyroCb-related data suggests that pyroCb in southeast Australia can form in a variety of atmospheric conditions. These results may have important implications for forecasting pyroCb potential.

**Supplementary Information:**

The online version contains supplementary material available at 10.1038/s41598-025-22530-0.

## Introduction

Few wildfire-related phenomena have caught the attention of the broader wildfire and fire weather research communities like pyrocumulonimbus (pyroCb) have in the last decade or so. As wildfire-generated thunderstorms^1^, pyroCb are notorious for their unpredictability and for introducing thunderstorm-related hazards—such as cloud-to-ground lightning, damaging winds, and even fire-generated tornadic vortices—into what is often already a chaotic and dangerous wildfire environment^2–7^. These hazards make pyroCb potentially very dangerous for firefighters, other emergency personnel, and civilian populations in their vicinity.

PyroCb have become increasingly common in Australia, with southeast Australia most frequently impacted^8^. Of the 144 recorded pyroCb events in the Australian PyroCb Register (dating back to the start of the satellite record), 135 have occurred since 2003, with 45 recorded during the 2019–20 fire season alone^8^. Though they have been associated with some of Australia’s deadliest and most destructive fire-related catastrophes, including Ash Wednesday in 1983^8–10^, Black Saturday in 2009^2,11,12^, and most recently throughout the 2019–20 Black Summer^6,13^, the underlying processes associated with pyroCb have only relatively recently become a major focus of research. Given the hazards they pose, understanding atmospheric conditions associated with pyroCb development and the implications of such conditions on their behaviour is extremely important.

### Factors influencing pyroCb development

Peterson, et al.^5^ used a hybrid exploratory statistics- and case study-based approach to develop a conceptual model for pyroCb development in western North America that focused on both surface and atmospheric conditions. They found that pyroCb environments were often characterized by warm, dry, and windy conditions within a tall boundary layer and unstable, relatively moist conditions just above and within the entrainment layer—often in the mid-levels (700–500 hPa). Moisture within the entrainment layer has been linked to pyroCb development^6,7,14^.

At this time, no equivalent conceptual model is known to exist for any region in Australia. However, a number of studies, of Australian bushfire and pyroCb events—including Ash Wednesday (1983)^9,10^, Canberra (2003)^3,15,16^, Black Saturday (2009)^2,11,12,17^, Forcett-Dunalley (2013)^18,19^, Sir Ivan Dougherty (2017)^20,21^, and the Australian New Year Super Outbreak (2019–20)^6^, among others^22–24^—have lent substantial insights into atmospheric features commonly associated with Australian pyroCb, while underscoring the variable conditions in which they develop. Similar to western North America, boundary layer conditions in the lead up to Australian pyroCb tend to be warm-to-hot, relatively dry, and often windy. Conditions above the boundary layer are often moister, though the degree of mid-level moistening, the presence or lack of mid-level dry intrusions, and the degree of low-level moistening have varied considerably in the aforementioned case studies.

**Fig. 1:**
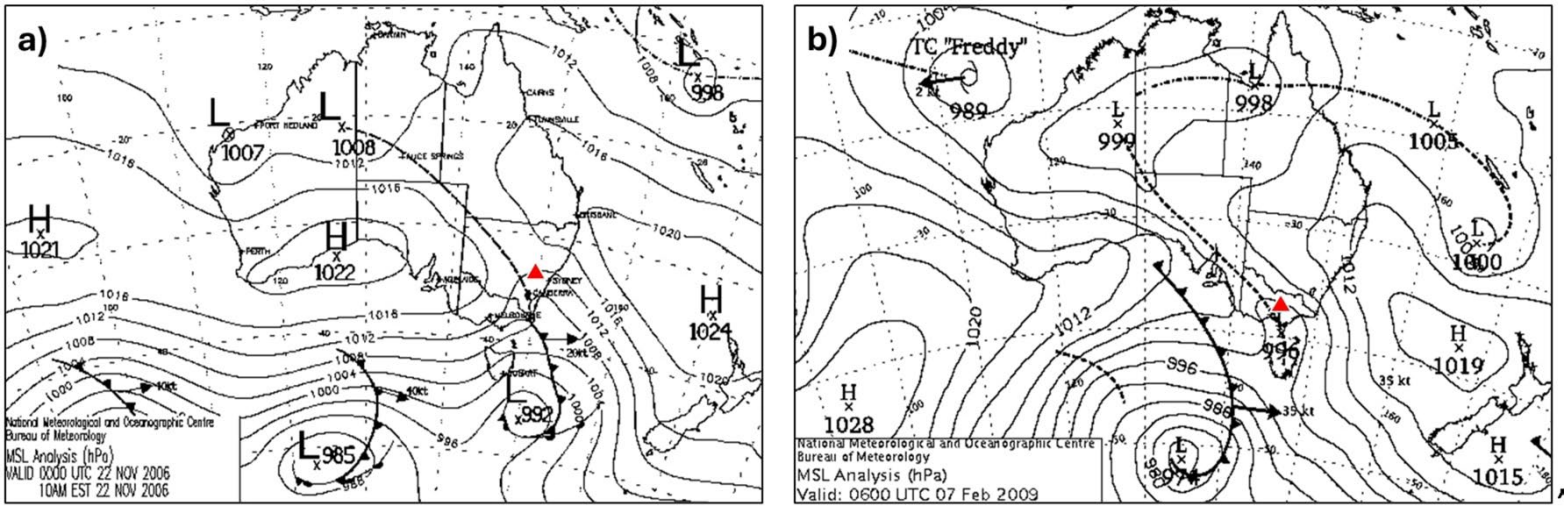
Synoptic charts shortly before two Australian pyroCb events (approximate fire locations are denoted by the red triangles): (**a**) the Grose Valley fire in Blue Mountains National Park and (**b**) the Kilmore East fire from Black Saturday.

One notable meteorological variable among Australian pyroCb cases is the role played by surface fronts, troughs, and other boundaries in pyroCb initiation. All of the aforementioned cases were characterized by warm air advection within the warm sector of a surface cyclone ahead of an approaching surface boundary. In some instances, such as the Canberra, Blue Mountains and Wollemi National Parks (Fig. 1a), and Forcett-Dunalley fires, pyroCb developed and dissipated entirely within the warm sector. In others, like the Murrindindi and Kilmore East fires on Black Saturday (Fig. 1b) and the Sir Ivan Dougherty fire, pyroCb initiated upon the arrival of a surface boundary and wind change that introduced cooler, moister conditions to the fireground, significantly modifying the low-level portions of the atmospheric profile and likely introducing surface-based instability^20,21^.

Wind speed may also be an important but potentially paradoxical variable in pyroCb development. Several catastrophic Australian bushfire and pyroCb events have occurred under windy conditions (e.g., Ash Wednesday, Canberra, Black Saturday)—in both the pre-^24^ and post-boundary regimes^2,10–12^. However, high low-level winds may inhibit pyroCb potential. The Pyrocumulonimbus Firepower Threshold (PFT)^25^, adapted from Briggs’s plume model^26^, estimates the minimum total fire power (heat flux) necessary to induce deep moist convection (pyroCb). One of its major components is mixed-layer wind speed. Because stronger winds may tilt the fire’s plume, enhance entrainment of cooler, more stable air, and reduce plume buoyancy, they result in a higher PFT^25,27,28^. Conversely, higher wind speeds often lead to increased fire size, generating stronger convective updraughts^29,30^. This wind-related paradox is highlighted by Badlan et al.^29^ in their simulations of violent pyroconvection. They found that fires under lower-wind conditions generated stronger updraughts than fires of the same size under windier conditions. However, they also found the updraughts from larger fires were significantly stronger than those generated by smaller fires under the same wind regime.

Speed and directional wind shear may have a similarly confounding impact on pyroCb potential and behaviour, as strong vertical shear has been tied to tilted and less buoyant plumes^31^. While North American pyroCb environments have tended to be characterized by weak shear, notable exceptions exist^32^. Australian pyroCb events have occurred in a wide variety of wind shear regimes, including weak-to-moderate unidirectional^3,4^, strong unidirectional^19^, moderate unidirectional undercut by a backing near-surface southwesterly change (resulting in a potentially supercellular pyroCb^20^), and strong directional shear, with veering winds aloft undercut by a backing near-surface change^33^.

Though our focus is primarily meteorological, other factors potentially contributing to enhanced fire spread and intense pyroconvective updraughts should be acknowledged. These include fuel-related factors (type, moisture, load) and dynamic wind/fire/terrain interactions (e.g., vorticity-driven lateral spread^34,35^, uphill fire runs^29^). They often act in concert, resulting in large areas of deep flaming, which in turn generate powerful updraughts. Notably, roughly symmetrical flaming regions appear more efficient at producing strong updraughts than narrow, linear fire fronts of the same area^29,36^. The localized nature of these factors makes them difficult to account for when broadly assessing pyroCb potential. However, efforts to account for the fire’s contribution to destabilization (through heat flux and/or moisture flux from combustion of fuels) have been undertaken, though to what degree is debated^25,28,37,38^.

### Statistical analyses of Australian pyroCb and non-pyroCb wildfire environments

Several prior studies have brought together numerical weather prediction model-derived data for large numbers of pyroCb and non-pyroCb (standard) wildfire events in southeast Australia^39–42^. These studies primarily used fire danger and fire weather indices as representatives of meteorological conditions—often FFDI and/or Fuel Moisture Index (FMI)^43^ for near-surface conditions and the Haines Index^44^ or Continuous Haines Index (C-Haines)^42^ as a combined indicator of lower-to-mid-level atmospheric stability and dryness. Studies have shown lower-to-mid-level stability to have a stronger correlation with pyroCb development than surface-based weather conditions alone^39–41^. Ma et al.^40^ demonstrated through regression analysis that lower-to-mid-level stability is a better indicator of pyroCb potential than both lower/mid-level dryness and surface dryness. Wilson et al.^41^ found that while lower-to-mid-level stability is moderately correlated with surface dryness for standard wildfire events, the relationship is much stronger for pyroCb events. They cautioned, however, that high lower-to-mid-level instability has been associated with wide-ranging surface weather conditions.

While detailed case studies have provided useful insights into some of southeast Australia’s most impactful pyroCb events, they have also highlighted inconsistencies and collectively represent fewer than half the region’s recorded events. Furthermore, individual case studies have not included comparative analyses of standard wildfires—as Peterson et al.^5^ did when developing their western North American model—increasing the risk of confirmation bias and potentially obscuring important differences between pyroCb and standard wildfire-associated atmospheric environments. Taking a step back to analyse the bigger picture offers a sensible supplement to individual case studies, and while the aforementioned statistics-based studies^40,41^ have made important contributions, their focus has been exclusively within the lower-to-mid-troposphere and on simple fire weather indices. Thus, it remains unclear what distinct thermodynamic or wind profile features may exist above the levels represented by those indices.

Here, we construct, examine, and statistically compare vertical temperature, dew point, and wind profiles associated with pyroCb and standard wildfire events in the southeast Australian mainland (Victoria, New South Wales, and the Australian Capital Territory) over the 30-year period from 1991 to 2020. We also calculate lapse rates and precipitable water vapour values for different layers within the atmospheric column and identify several important differences between pyroCb- and standard wildfire-associated profiles. The atmospheric profiles explored here have significant implications for fire weather forecasting, as they provide a statistically rigorous foundation for enhanced understanding of the meteorological drivers of pyroCb in southeast Australia. Furthermore, atmospheric profiles can offer insight not only into pyroCb potential, but also into potential hazards and behaviours of pyroCb once they form.

## Data and methods

Our approach draws upon prior efforts to characterize the surface and atmospheric conditions conducive to pyroCb development^5,40,41^ and combines aspects of the different methodologies that were employed in those works.

### Wildfire, pyroCb, and atmospheric data

A dataset of standard wildfires and pyroCb events in southeast Australia was compiled by Ma et al. ^40^ from the bushfire history databases for New South Wales (including the ACT) and Victoria ^2,3^, and the Australian PyroCb Register^8^. The dataset, which spans the period 1980–2020, includes standard wildfires exceeding 10 hectares and all pyroCb events in NSW, the ACT, and Victoria, regardless of the area burnt. However, to better identify the distinguishing features of favourable pyroCb environments on days with conditions likely to favour significant fire activity, large standard wildfires (≥ 1000 ha final burnt area) were chosen as a reference. We therefore focused our analyses on the subset containing only standard wildfires over 1000 hectares plus all pyroCb events from 1991 to 2020, as was used by Wilson et al.^41^, along with two additional pyroCb events from 2020 (added to the Australian PyroCb Register in 2023). The resulting dataset consists of 94 pyroCb events and 986 large standard wildfire events.

Hourly estimates of surface and atmospheric weather variables at various pressure levels for each event were sourced from the ERA5 global reanalysis dataset^47^. ERA5 provides data over a 0.25° latitude × 0.25° longitude grid for variables at atmospheric levels from the surface up to 3 hPa. It offers an alternative to relying solely on observed surface and radiosonde data, which in Australia may be recorded at locations far from the event sites and collected only once or twice daily. ERA5 data have been independently validated for Australia^40,48,49^, with strong performance across key variables. ERA5 near-surface wind speed has shown relatively lower correlation with observations^48^ but remains appropriate for large scale analysis when interpreted in context.

An unfortunate limitation of both standard wildfire history data and pyroCb data in Australia (and for much of the rest of the world) is that information regarding timing of events is inconsistent and therefore missing for the vast majority of events in the combined southeast Australian dataset. Thus, rather than focusing on conditions at the exact time of pyroCb initiation, we considered the conditions throughout the afternoon and early evening hours of pyroCb event days at the locations where pyroCb occurred and then compared them to the atmospheric conditions associated with standard wildfire events.

Data were gathered at 27 pressure levels (1000 to 100 hPa) and near the surface for five basic variables: temperature, dew point temperature, relative humidity, and the U- (west/east) and V- (south/north) components of wind. The U- and V-components were used to calculate wind speeds and directions at each level. A filter was applied, based on surface pressure, so that only pressure levels above the terrain surface were considered. This process was repeated for each of the times of interest—00, 04, 06, and 08 UTC (10:00, 14:00, 16:00, and 18:00 Australian Eastern Standard Time, respectively). 00 UTC was chosen as it is around the time when rawinsonde-containing weather balloons are often launched at various sites across eastern Australia, so it is a common forecasting reference. 04, 06, and 08 UTC were chosen as they typically represent the peak of fire weather conditions and the timeframe during which many impactful front/trough passages/cool changes and pyroCb events have been documented^6,10,11,20,39,50,51^, though there have been exceptions^6,11,24,52^. While this approach may not always capture peak fire conditions for standard wildfire events, it represents the best available method given the limitations of the fire history databases and provides a robust dataset well-suited for statistical analysis.

### Construction of median atmospheric profiles and statistical testing

For each of the four times of interest (00, 04, 06, and 08 UTC) median, first quartile (Q1), and third quartile (Q3) values of temperature, dew point, relative humidity, and wind speed were calculated. This provided an overview of atmospheric profiles associated with both pyroCb and standard wildfire event days, henceforth referred to as the median atmospheric profiles. To visualize the median atmospheric profiles, median temperature and dew point values for both groups, along with their inter-quartile ranges (IQR), were plotted together on skew-T/log-P aerological diagrams for each of the four times of interest. Although the diagrams offer only a generalized overview of atmospheric conditions, being able to visualize the median profiles highlights key differences between the pyroCb and standard wildfire groups. This is true not only for difference in variable values between the two groups, but also with respect to understanding the general relationship between the median temperature and dew point lines (the profile “shape”) for each event type, the spread of the variable values (represented by the IQR), and how/whether these profile characteristics vary across the four times of interest.

Observations of the differing profile shapes across time also prompted a more in-depth look into several additional parameters that could easily be calculated from the median profiles—low-level (surface-to-3 km), mid-level (700-to-500 hPa), and total (surface-to-100 hPa) precipitable water and low-level and mid-level lapse rates. Precipitable water is the amount of water that would be present if all water vapour in a given atmospheric layer within a vertical column were saturated and measured^53^, while the lapse rate is the rate at which the temperature changes throughout a given layer of the atmosphere, with low- and mid-level lapse rates commonly used in thunderstorm forecasting.

Simple statistical testing was performed to determine whether differences observed in the median profiles were statistically significant. Although much of the data were normally distributed with approximately equal variances, this was not always true. Therefore, to maintain consistency, a series of Mann–Whitney *U*-tests^54^ were performed for temperature, dew point, relative humidity, and wind speed at each time and at nine selected levels (200, 300, 400, 500, 600, 700, 850, 950 hPa, and near-surface) as well as for the five additional precipitable water and lapse rate measures across the four times of interest.

Even in instances where the shapes and/or spread of two distributions are different, the Mann–Whitney *U*-test is still quite powerful, as its test statistic (*U*) can easily be converted to an effect size, which approximates the probability of a randomly selected value from one group being greater than a randomly selected value from a second group (i.e., it is a probability of superiority)^55^. For each test here, the null hypothesis was that when randomly selecting a value from both the pyroCb and standard wildfire groups, the probability of the pyroCb group’s value being larger was equal to the probability of the standard wildfire group’s value being larger; $Prob\left(P>S\right)=Prob(P<S)$, simplified as $P\approx S$.

For each test, a result ($P>S$, $P<S$, or $P\approx S$) was based on which group had the larger mean ranks (accounting for ties) and whether the two-tailed *p*-value indicated the difference between groups was statistically significant ($p<.05$). For statistically insignificant results, we were unable to reject the null hypothesis ($P\approx S$). Though the Mann–Whitney effect size has many names in the statistics literature^55,56^ and several slightly different methods for calculating it, they all describe the same parameter and show the probability of superiority described previously. Here, the effect size (ES) was calculated as:

1 $$ES=1-\left(\frac{{U}_{S}}{{n}_{1}{n}_{2}}\right).$$

${U}_{s}$ is the reported *U*-value (the smaller of the two possible *U*-values) and ${n}_{1}$ and ${n}_{2}$ are the sample sizes of the pyroCb and standard wildfire groups, respectively. ES ranges from 1.0 (most substantial) to 0.5 (least substantial). ES was only calculated for statistically significant results; for insignificant results, it should be interpreted as $\text{ES}\approx 0.5$. Finally, wind direction and speed were also explored through the creation of a series of wind roses for each of the four times at five selected levels (200, 500, 700, 850 hPa, and 10 m above ground level).

## Results

### Temperature and moisture

**Fig. 2:**
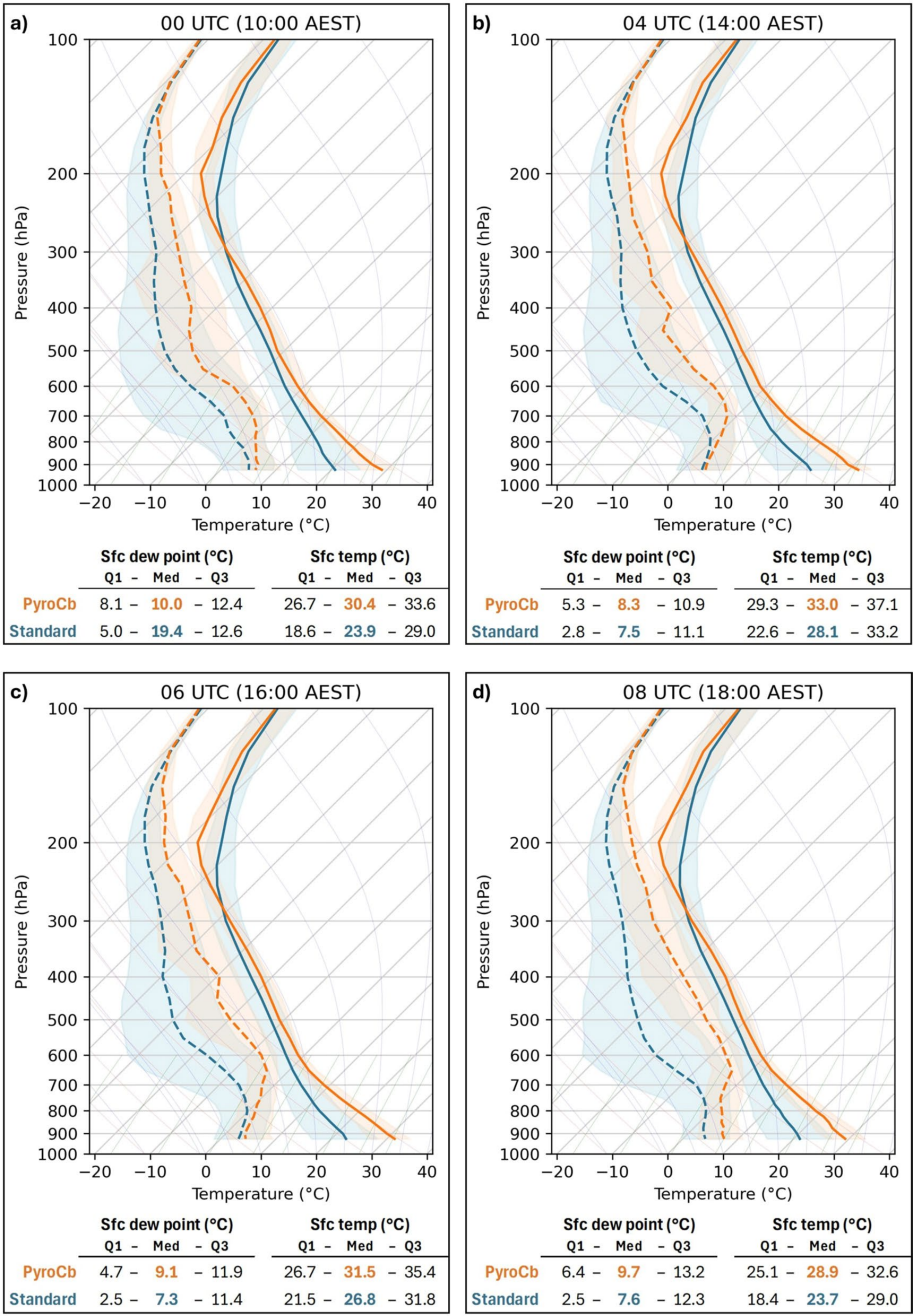
Median atmospheric profiles of temperature (solid) and dew point (dashed) for pyroCb (orange) and large standard wildfire (blue) groups, 1991–2020: (**a**) 00 UTC, (**b**) 04 UTC, (**c**) 06 UTC, and (**d**) 08 UTC. Interquartile ranges are shaded. Full statistical test results are in Supplementary Tables S1 (temperature), S2 (dew point), and S3 (relative humidity).

Figure 2 displays the median temperature and dew point profiles for 00, 04, 06, and 08 UTC on a series of skew-T/log-P aerological diagrams. Data were plotted down to 925 hPa, the lowest level to be above the surface for more than half of both pyroCb and standard wildfire cases across all four times. Several tendencies can be seen in the shapes of the median pyroCb and standard wildfire group profiles. First, while both groups were characterized by generally warm-to-hot surface temperatures and low surface dew points, the pyroCb profiles were much warmer in the lower levels, with much less spread in temperature and dew point values and minimal overlap in IQRs for the temperature profile from below about 700 hPa. Both classes of profiles are indicative of well-mixed boundary layers—particularly from 04 to 08 UTC. However, the pyroCb group profiles show an obvious, common occurrence of hot, dry surface conditions, with very deep mixed layers that take on a classic inverted-V pattern—a hallmark of high fire danger conditions in southeast Australia (prior to any pre-frontal trough/cold front passages). At the top of the inverted-V, the relative humidity tends to rise, as indicated by the median temperature and dew point lines drawing together. Both profile types show a general moistening in this region throughout the day, but it is especially pronounced in the pyroCb group profiles, where the column from the top of the inverted-V through the mid-levels and even into the upper levels (above 500 hPa) becomes substantially moister by 06 and 08 UTC compared to 00 and 04 UTC. It should be noted that there is considerable spread in dew point values throughout both profile types across time, with occasional dry to extremely dry layers being evident in the median standard and pyroCb group dew point lines and further evidenced by the shapes of the IQRs, especially the Q1 values. On pyroCb event days, these seem to most frequently occur around 500 hPa and again at around 350 hPa.

Statistical test results indicate that most of the observed temperature differences between the groups are significant (Supplementary Table S1). The pyroCb group temperature is significantly warmer than the standard wildfire group at all levels below 400 hPa across all times examined, with especially large differences from 700 hPa to the surface. Effect size (ES) values are consistently high in this layer (ranging from 0.766 to 0.845), peaking just below 900 hPa. Notably, even at 00 UTC (10:00 AM AEST), the median 2-m and 850-hPa temperatures for the pyroCb group are 34.0 °C and 22.0 °C, respectively, compared to 22.1 °C and 15.2 °C, for the standard wildfire group. These warmer low-level temperatures for the pyroCb group were the greatest differentiator between the two groups.

**Fig. 3:**
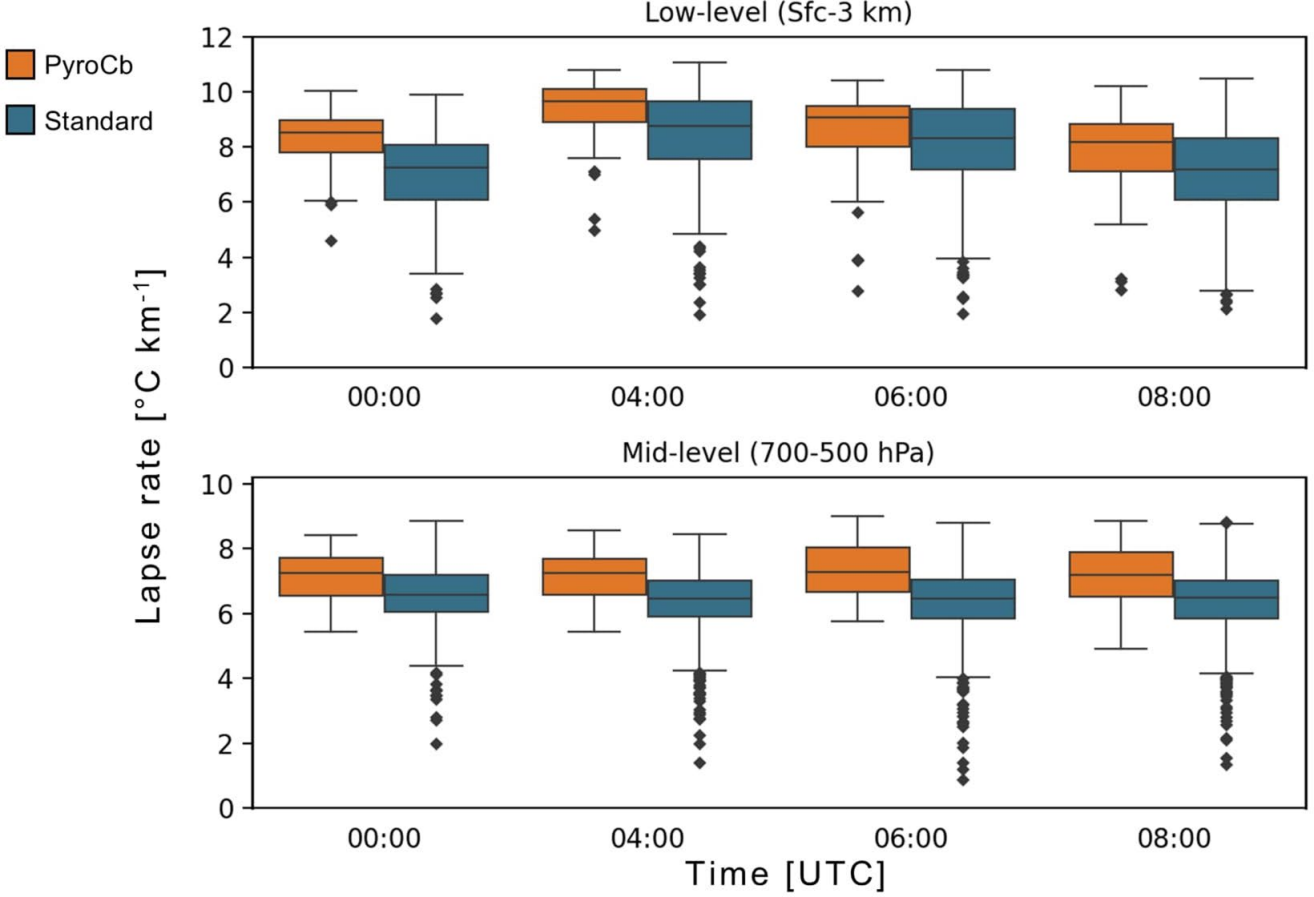
Lapse rates for pyroCb (orange) and large standard wildfire (blue) groups, 1991–2020. Full statistical test results are in Supplementary Table S4.

Above 700 hPa, ES values notably decrease with height, reflecting the diminishing temperature contrast between the two groups with height. However, near the 200-hPa level, ES values again increase due to the tendency for the tropopause to be higher for pyroCb events. Results (Fig. 3) confirm that both low-level and mid-level lapse rates are significantly steeper for the pyroCb group across all four times. Low-level lapse rates for pyroCb days tend to be extremely steep, even as early as 00 UTC (10:00 AEST), compared to the standard wildfire group (ES = 0.781). The median value for low-level lapse rate peaks at 9.6 °C km^−1^ by 04 UTC, indicative of unstable low-level conditions (with near dry- to-super-adiabatic lapse rates) being common in the boundary layer during peak heating on pyroCb days. However, median low-level lapse rates for the standard wildfire group also become quite steep by 04 UTC, thereby reducing the ES to levels below 0.700 for the test results from 04 UTC onward. With the exception of 00 UTC, the differences in lapse rates between the two groups is even more pronounced in the mid-levels, where the ES for the mid-level lapse rate tests result ranges from 0.695 at 00 UTC to 0.767 at 06 UTC. Furthermore, the pyroCb group’s median mid-level lapse rate value is greater than the Q3 value for the standard wildfire group across all four times, further indicating that mid-level lapse rates are a strong differentiator between the two event types. Interestingly, the median values for the mid-level lapse rates for both groups hardly changed throughout the day, ranging from 7.2 to 7.3 °C km^−1^ for the pyroCb group and from 6.5 to 6.6 °C km^−1^ for the standard wildfire group.

The test results for dew point and relative humidity also reveal several significant differences between the two groups. First, while the median dew point value for the pyroCb group is higher than for the standard wildfire group for every level at every time examined, the significance of these differences varies from highly significant, with ES in the mid-0.6 s to mid-0.7 s above 700 hPa, to less significant and ES near 0.600, to insignificant altogether at and below 850 hPa. This might be due to the large spread in dew point values for both groups observed in the aerological diagrams as discussed earlier. The exception is at 08 UTC, where the significance of dew point test results and ES are relatively higher, even in the lower levels. By contrast, relative humidity results paint a much clearer and more consistent picture, with pressure levels above the 700-hPa level having higher relative humidity for the pyroCb group, while those below this level tend to significantly lower relative humidity values for the pyroCb group. These findings are apparent in the median profiles across all four times, as the dew point depressions are smaller in the mid-levels for the pyroCb group but then much larger nearer the surface. The increase in median relative humidity values at 600 hPa for the pyroCb group is especially noteworthy, as it jumps from around 31% at 00 UTC to 70% by 06 UTC. During the same time period, the median values for the standard wildfire group remain very near or just below 30%. Thus, the ES during the same time period increases markedly from 0.570 at 00 UTC to 0.704 by 06 UTC. A similar pattern is observed at 500 hPa but to a slightly lesser extent.

**Fig. 4:**
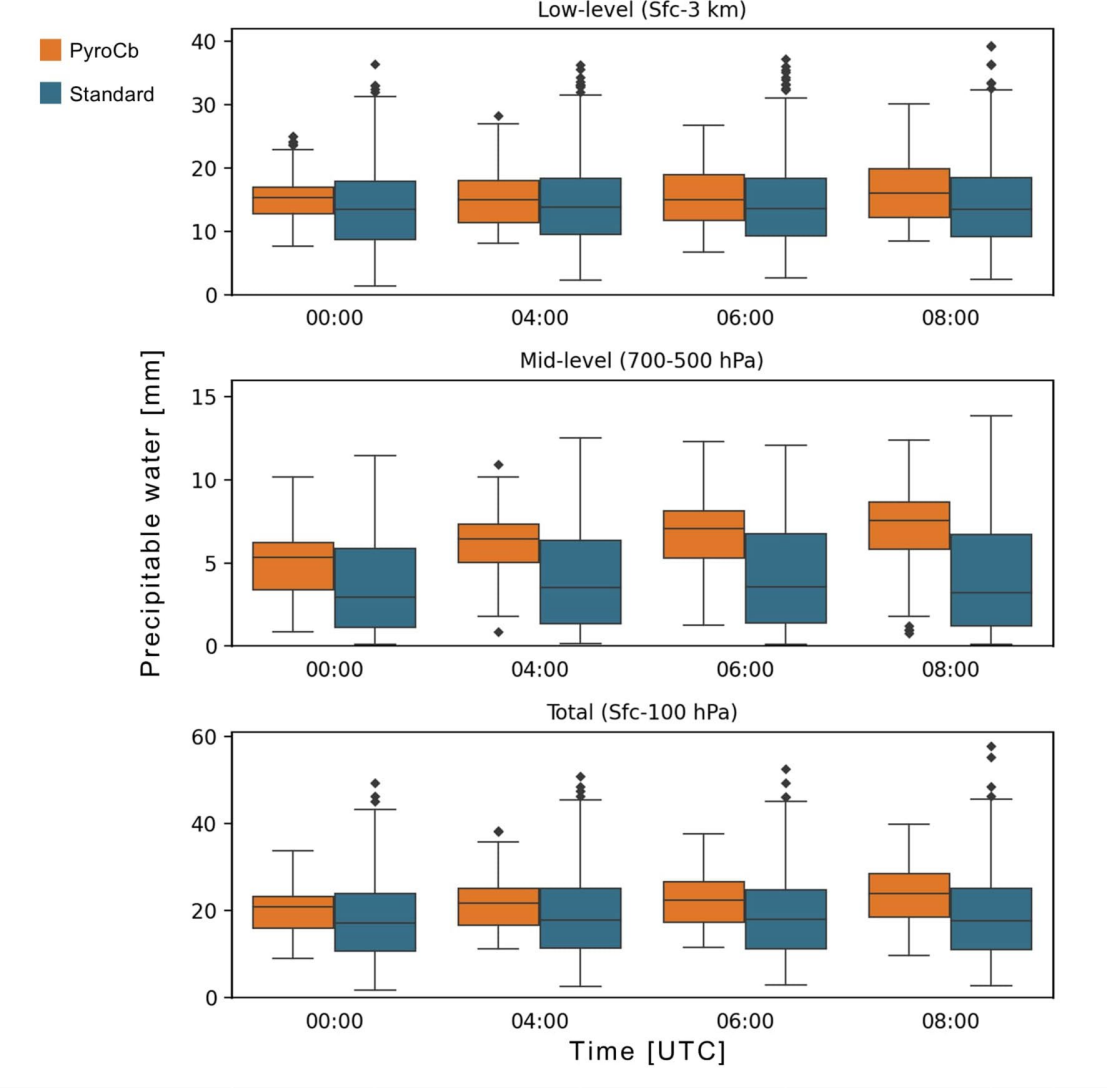
Precipitable water for pyroCb (orange) and large standard wildfire (blue) groups, 1991–2020. Full statistical test results are in Supplementary Table S4.

At 500 and 600 hPa, both dew point and relative humidity are significantly greater across all four times for the pyroCb group, a very strong indicator that the total moisture content within this region is likely substantially higher for the pyroCb group. Precipitable water calculations and their statistical test results indicate that while differences between the two groups for all three of low-level, mid-level, and total precipitable water were statistically significant across all four times measured, the most significant differences (highest ES values) were for the mid-levels (Fig. 4). Unlike mid-level lapse rates, where little change was observed across time, mid-level precipitable water values trend upward throughout the day, with median pyroCb group values jumping from 6.3 mm at 00 UTC to 8.7 mm at 08 UTC. ES values also increase to above 0.700 for the three later times, thus making mid-level precipitable water another strong differentiator between the pyroCb and standard wildfire groups.

### Wind speed and direction

**Fig. 5:**
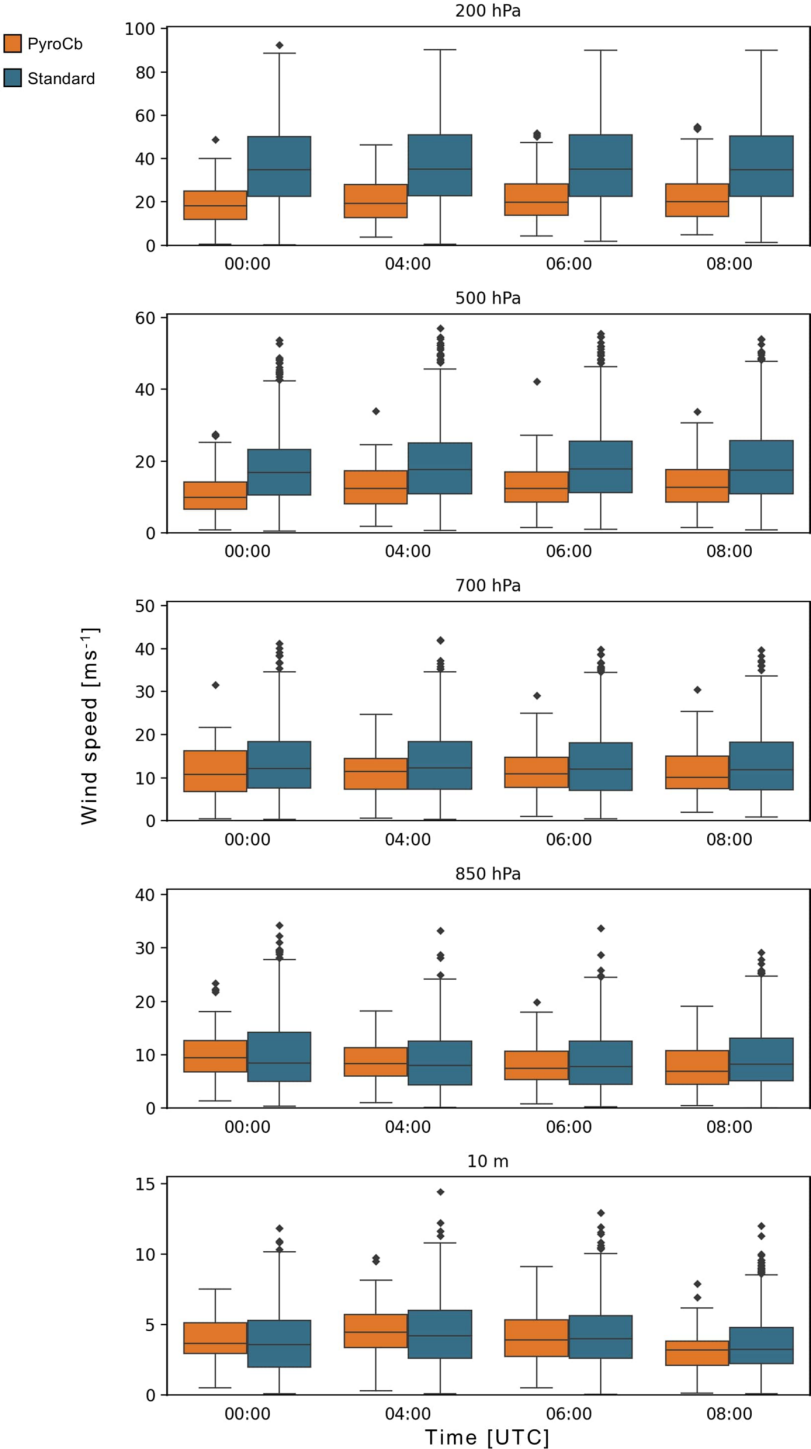
Wind speed at select pressure levels for pyroCb (orange) and large standard wildfire (blue) groups, 1991–2020. Full statistical test results are in Supplementary Table S5.

Analysis of wind speeds associated with the median profiles also revealed several differences between the two groups. Upper-level wind speed for the pyroCb group tends to be significantly lower than for the standard wildfire group across all times (Fig. 5). The magnitude of this difference increases with height, evidenced by the very high ES values (> 0.700) for every level at or above 400 hPa for every time (except for 400 hPa at 08 UTC). This makes upper-level winds one of the biggest differentiators between the pyroCb and standard wildfire groups. However, with a few exceptions, differences in wind speed in the mid- and lower levels mostly become insignificant. In general, the pyroCb group’s median values are slightly lower, but with large variability in wind speeds in both groups. As for the wind speeds themselves, they tended to be rather low, with pyroCb group median values at 10 m ranging from 3.3 m s^−1^ to 4.1 m s^−1^ (12.0 km h^−1^ to 14.7 km h^−1^) and standard wildfire group values being slightly higher. Values in the lower levels above the surface tend to be slightly higher, with median speeds for both groups throughout this region ranging from about 6.5 m s^−1^ to 10.0 m s^−1^, with the highest speeds at 00 UTC. Above the boundary layer, median wind speeds tend to increase markedly with height, particularly in the standard wildfire group.

**Fig. 6:**
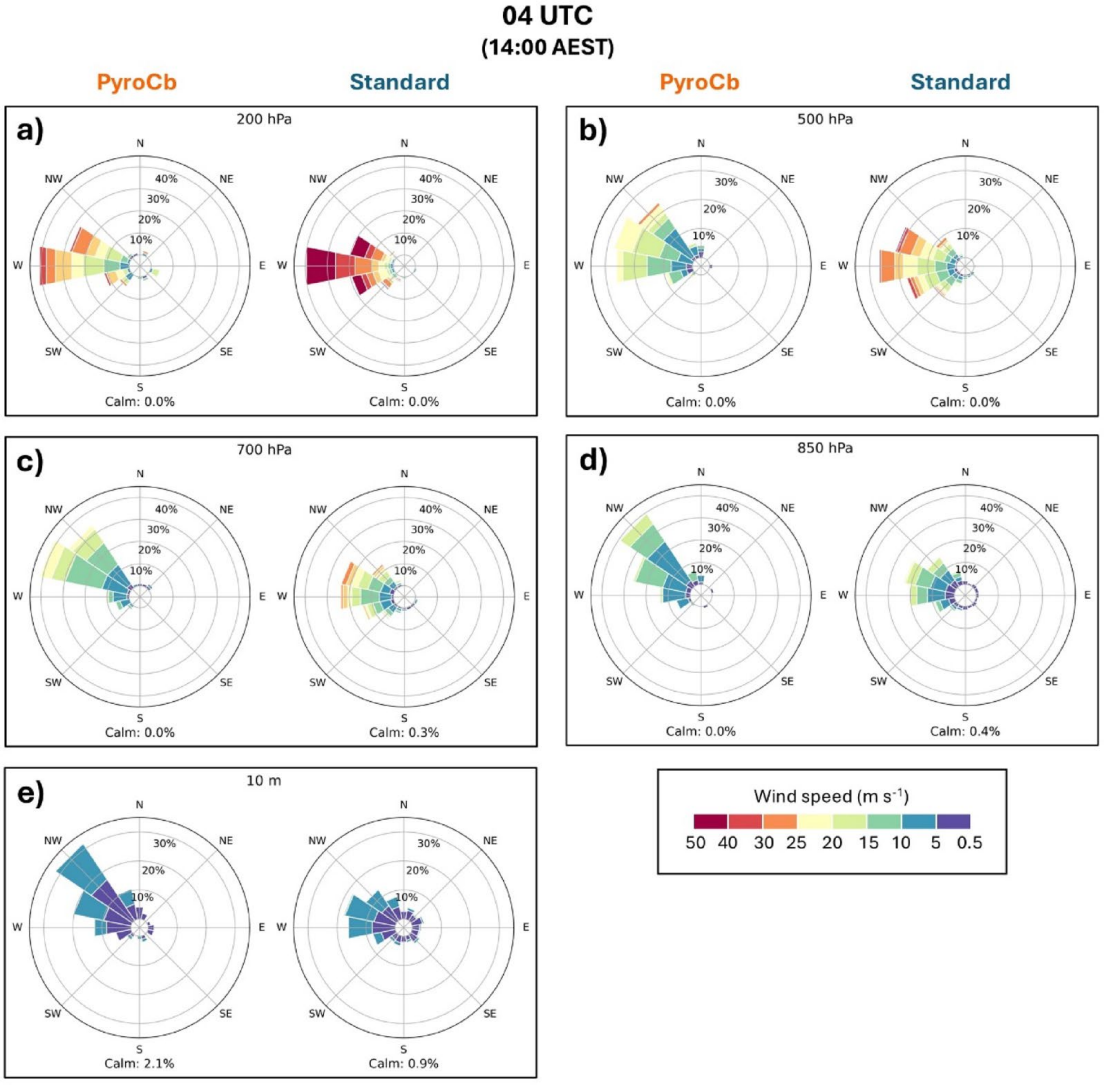
Wind roses for 04 UTC for pyroCb and large standard wildfire groups, 1991–2020: (**a**) 200 hPa, (**b**) 500 hPa, (**c**) 700 hPa, (**d**) 850 hPa, and (**e**) 10 m above ground level. Note the radial axes values are not uniform across all levels and are based on data distribution. Wind roses for 00 UTC and 06 UTC are in Supplementary Figs. S1 and S2. Full statistical test results for all times are in Supplementary Table S5.

**Fig. 7:**
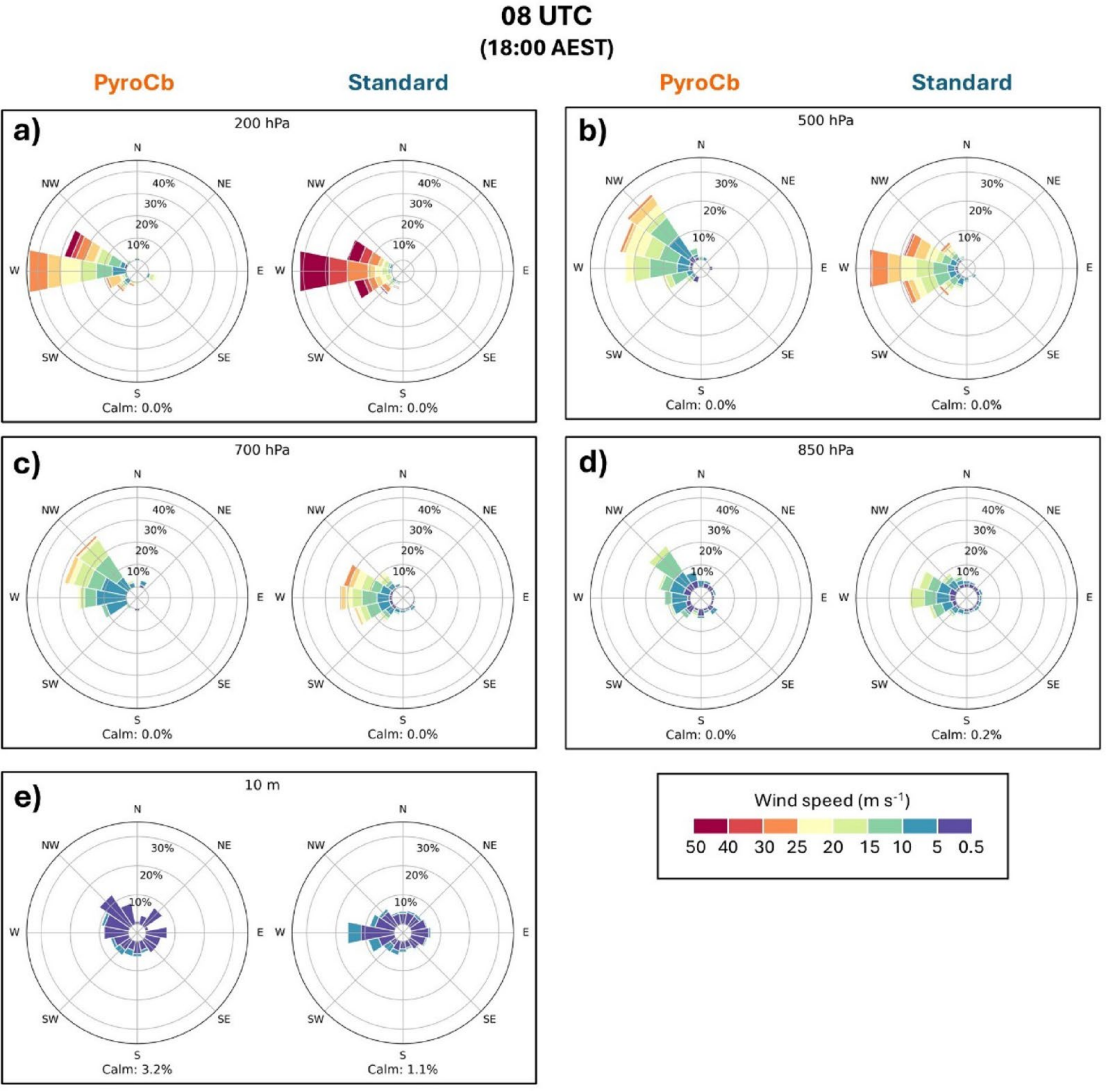
As in Fig. 6, but for 08 UTC.

Wind direction (along with speed) for each event in the southeast Australian dataset is plotted on a series of wind roses at select levels within the troposphere and based on event type (Figs. 6 and 7). While no statistical testing was performed for wind direction, a few noteworthy tendencies are seen in the wind roses. One is that the two groups share similar wind regimes but with a few exceptions: the aforementioned difference in wind speed throughout the upper levels, a slightly more northward component being more common for the pyroCb group across all times, and more spread among the standard wildfire group. Second is the general veering of the winds with height evident in both groups, with the northerly near-surface winds consistent with low-level warm air advection and generally zonal upper-level flow. This pattern is especially evident in the pyroCb group, with the most common wind direction being north-northwest at 10 m and becoming west by 200 hPa. As with the thermodynamic profiles, front and/or trough passages—which often come with a west or southwest wind change in the lowest levels—are not obvious in either group for most times. However, 10-m winds give some indication that diurnal changes in direction are fairly common during pyroCb event days. At 04 UTC, well over 50% of pyroCb event days had a northwesterly wind (north-northwest, northwest, or west-northwest), while southwesterly and southerly winds (ranging from west-southwest to south-southeast) accounted for well under 10% of the total. However, by 08 UTC (Fig. 7), northwesterly winds accounted for only about 30% of the total, with southwesterly and southerly winds increasing to roughly 25%. A similar backing trend is visible for the standard wildfire group, though there is more variability by 08 UTC.

## Discussion and conclusion

This study has identified several clear and statistically significant differences between the atmospheric environments associated with pyroCb and standard wildfire events in southeast Australia. Most notably, pyroCb event days are often characterized by diurnal mid-level moisture advection—a feature much less common during standard wildfire events—that likely enhances deep convective potential^5,7,14^. Additionally, pyroCb environments tend to have warmer, drier low-level conditions, steeper mid-level lapse rates that are often established by at least late morning, and lighter mid-to-upper-level winds. These results build on the insights from numerous southeast Australian pyroCb case studies and have provided a multi-decade, profile-based statistical confirmation of key atmospheric features favouring pyroCb formation in the region.

Findings are largely consistent with those in western North America, with near-surface conditions favourable for fire spread while moistening mid-levels in the plume’s entrainment region being conditionally favourable for high-based deep convective development^5,14^. Southeast Australian pyroCb events tend to have warmer near-surface conditions than their North American counterparts, but relative humidity values are similar across the two regions. Both the pyroCb-producing and standard wildfires tend to be associated with veering wind profiles and similar low-level wind speeds, but pyroCb events are more likely have lighter mid- and upper-level winds. Altogether, these results are consistent with the pre-boundary warm sector environments described in (or inferred from) numerous case studies of Australian pyroCb events, including the Canberra^16^, Wollemi/Blue Mountains National Parks^24,52^, Black Saturday^2^, Forcett-Dunalley^18,19^, and Sir Ivan fires.

The observed pattern of mid-level moisture advection on pyroCb event days stands in stark contrast to the consistent (and lower) amounts of mid-level moisture associated with standard wildfire events. Relatively moist mid-levels increase the potential for moist air entrainment into a fire’s plume just above the mixed layer, along with subsequent condensation and latent heat release—ultimately leading to a more buoyant and deeper convective plume^5,7,14^. Thus, to see the amounts of mid-level moisture increase so greatly throughout the afternoon hours (during peak surface fire spread conditions) is very significant. Further research is necessary to determine the source and atmospheric processes responsible for this phenomenon.

Conversely, steep mid-level lapse rates, along with warm low-level conditions are often present for the duration of the 00 to 08 UTC timeframe. For many events, the inverted-V pattern within the profile becomes established from at least 04 UTC. The time period from 04 to 06 UTC appears to have the greatest overlap of both favourable fire spread conditions at the surface (highest low-level temperatures, steepest low-level lapse rates, and the lowest low-level relative humidity) and favourable conditions for high-based thunderstorms above the boundary layer (steep mid-level lapse rates and increasing mid-level moisture—both in terms of relative humidity and precipitable water). Compared to the temperature profiles, there is substantially more variation in dew point profiles, with occasional dry intrusions (like those seen in the Black Saturday^2,20^ and Sir Ivan events) evident in the mid- and upper levels, further indicating that pyroCb in southeastern Australia may form in a variety of moisture regimes.

The most significant limitation to this study was the quality and availability of data within the state fire histories databases and the Australian PyroCb Register^8^—the sources of the combined pyroCb and standard wildfire dataset analysed here. Relying solely on the starting date for the standard wildfires almost certainly did not always capture the conditions that fires were under when peaks in fire activity occurred. Unfortunately, for nearly all fires, this was the only date available. Furthermore, data on fire spread are simply not available for the vast majority of wildfires. This likely partially explains the larger variability in the profiles of the standard wildfire group. However, it also helps to highlight the importance of the test results that were still not statistically significant or were significant but with relatively low ES.

Secondly, not having more precise event times for the majority of pyroCb events was somewhat of a limitation, as pyroCb may form in dynamic environments—such as during a cold front/trough passage. These passages often introduce significant changes to the pre-existing atmospheric profile, but data from them could become lost in the median profiles, meaning results might mostly characterize pre-trough/front environments in such instances where a passage eventually occurred. While an important consideration, several significant differences between the pyroCb and standard wildfire event groups remain. While the lack of timing information for the majority of pyroCb events forced the approach to be broadened, this turned out to be an unexpected advantage, as it revealed several differences—including the earlier deep mixing of the boundary layer for pyroCb event days, and the mid-level moistening trend that has occurred on numerous pyroCb event days—that would have been missed had only the life span of the pyroCb themselves (typically < 3 h) been considered.

Finally, the atmospheric profiles also raise some key questions and highlight areas of need for further research. First is whether the occurrence of very deep, well-mixed boundary layers is inherently favourable for pyroCb development or merely an impediment that many of the pyroCb were coincidentally able to overcome. It is possible that a very deep boundary layer could play both roles and is worth noting that conventional high-based thunderstorms in dry environments may form in similar meteorological conditions^57^. Secondly, while front and trough passages were not obvious in the median profiles, such passages and their temporal relation to several catastrophic pyroCb events in southeast Australia are well-documented^9–12,20^. Understanding the depth of a front/trough passage (several passages associated with notable pyroCb have been quite shallow^11,20^), along with the temperature and moisture gradients post-passage could have profound implications for the overall thermodynamic environment and pyroCb probability at a fire’s location. If near-surface dew point values increase much more rapidly than temperatures decrease, there could be a window of time where significant instability is introduced into the atmospheric profile even if the rest of the profile above the trough passage is unchanged, as likely occurred during the Sir Ivan pyroCb^20,21^.

Trough and front passages and their thermodynamic implications certainly warrant further research, as does determining which pyroCb events have been associated with them and which occurred independently, as these groups of events likely differ. However, while trough and front passages often substantially alter the lower levels of the atmospheric profile, the conditions leading up to their arrival likely play an important role in determining whether deep convective development ultimately occurs.

We found no statistically significant difference in wind speeds between the pyroCb and standard wildfire groups within the boundary layer, but the true reasons for this are likely complicated. First, as alluded to earlier, winds may play both a contributing and debilitating role in pyroCb potential, with its specific impact likely dependent on circumstances that impact plume updraught strength—such as the size and geometry of the active burning areas^29,36^ and the degree of plume tilt^25,28^. Secondly, wind data are very noisy, especially in the boundary layers on windy days and at the relatively low spatiotemporal resolution of ERA5^48^. This makes analysing low-level winds in any capacity come with a multitude of caveats. However, we feel confident that based on the analysis of mid- and upper-level winds, many Australian pyroCb tend to occur under moderate-to weak wind shear regimes, much like in western North America^5^. However, we again note that there have been notable catastrophic exceptions (e.g., Black Saturday^33^ and Forcett-Dunalley^19^). Overall, further research into the dynamic relationships between fire spread, fire geometry, plume tilt, and updraught strength could provide valuable insight into the apparent low-level wind paradox and help clarify how windy conditions influence pyroCb initiation.

Once a pyroCb has formed, understanding the wind profile it will exist in is extremely important, as the directions of the winds above the surface may play a key role in determining which direction (if any) the storm’s updraught will tilt, what locations will experience the effects of the pyroCb’s forward downdraught, what locations are most likely to experience pyroCb-generated lightning, and even potentially whether the pyroCb may take on supercellular characteristics, as was the case during the Sir Ivan pyroCb in 2017^20^. In both the Sir Ivan Fire and the Black Saturday^2^ events, thunderstorm updraughts tilted from northwest-to-southeast at an angle almost orthogonal to the surface wind change direction due to the mid- and upper-level winds. The median wind profiles examined here suggest that directional wind shear is a relatively common feature of pyroCb events in southeast Australia, even though it may not be particularly strong. However, we have only scratched the surface of the potentially large amounts of information that can be obtained from wind profiles. Thus, further research into atmospheric wind profiles and their impacts on pyroCb behaviour is needed. Given the common knowledge that not all thunderstorms are equal, we certainly shouldn’t expect fire-generated thunderstorms to be either.

Through the construction and analysis of median atmospheric profiles, we have shown that many of the conditions associated with pyroCb are significantly different from those commonly associated with large standard wildfire events. These differences specifically highlight an environment that is at least conditionally favourable for thunderstorm development, despite often very dry surface conditions. Furthermore, we have discussed some of the implications of the median profiles, including those associated with potential trough passages and wind shear. As pyroCb present a multitude of potential hazards for anyone near them and are becoming increasingly common in Australia, better understanding the atmospheric conditions associated with their development and how they may impact pyroCb behaviour continues to be extremely important.

## Supplementary Information

Below is the link to the electronic supplementary material.

Supplementary Material 1

## Data Availability

The data used to support the findings of this study are available from the corresponding author upon request.
