# Supplementary material for: Atmospheric profiles associated with pyrocumulonimbus in southeast Australia
Source: Sci Rep. 2025 Nov 4;15:38538. doi: 10.1038/s41598-025-22530-0 (PMC12586718; doi:10.1038/s41598-025-22530-0)

# **Atmospheric profiles associated with pyrocumulonimbus in southeast Australia**

Caleb S. Wilson | Jason J. Sharples | Jason P. Evans

## **Supplementary Information**

**
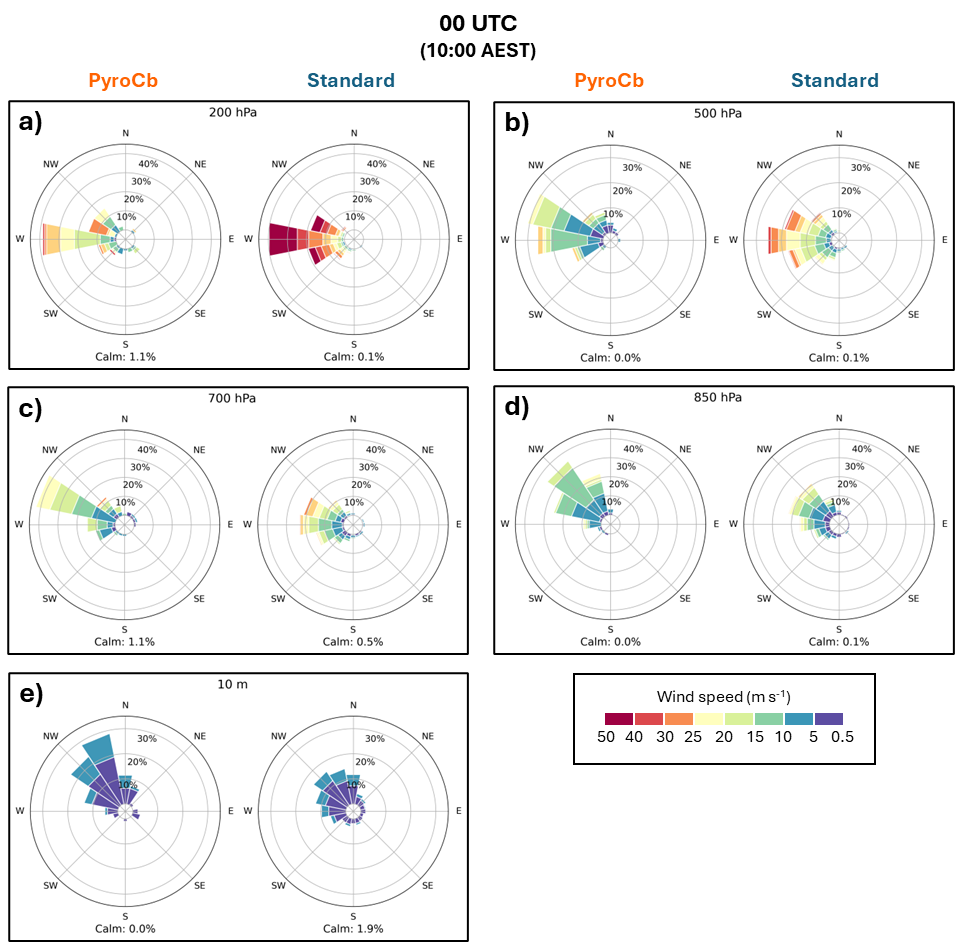
**

**Supplementary Figure S1.** Wind roses for 00 UTC (10:00 AEST) for pyroCb and large standard wildfire groups, 1991-2020: a) 200 hPa, b) 500 hPa, c) 700 hPa, d) 850 hPa, and e) 10 m above ground level. Note the radial axes values are not uniform across all levels and are based on data distribution.


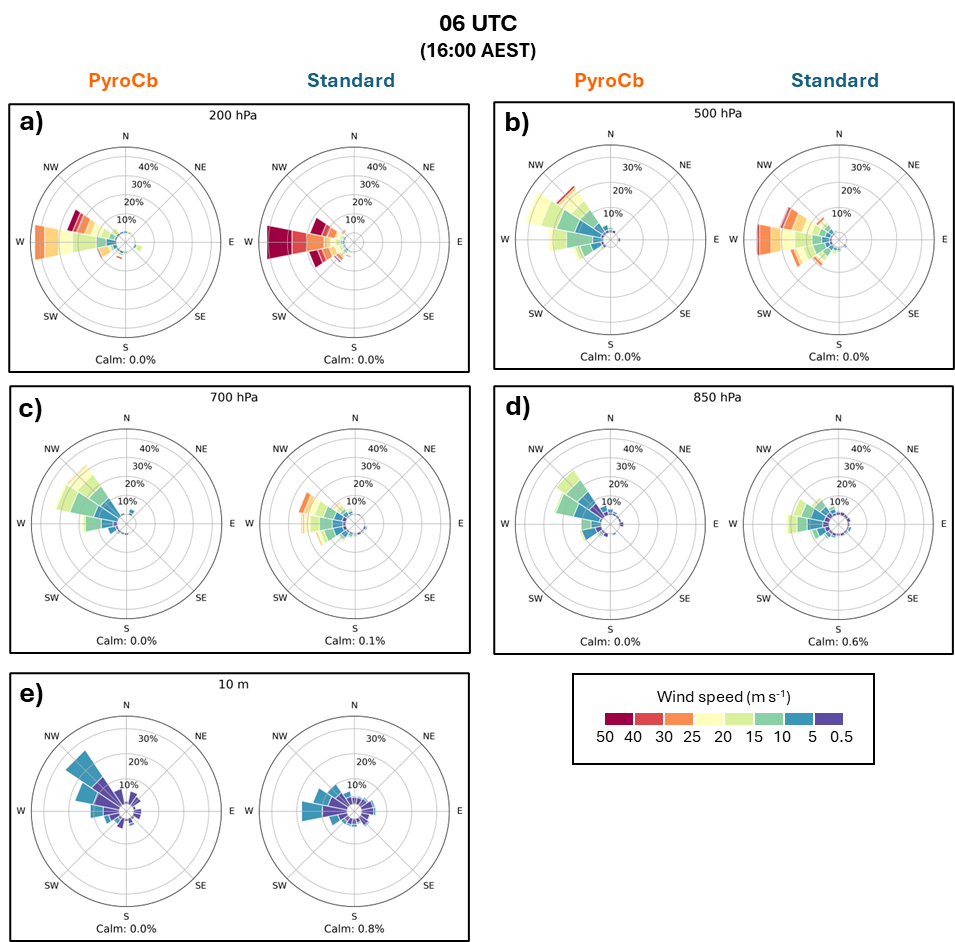


**Supplementary Figure S2.** Wind roses for 06 UTC (16:00 AEST) for pyroCb and large standard wildfire groups, 1991-2020: a) 200 hPa, b) 500 hPa, c) 700 hPa, d) 850 hPa, and e) 10 m above ground level. Note the radial axes values are not uniform across all levels and are based on data distribution.

**Supplementary Table S1.** Descriptive statistics and Mann-Whitney *U*-test results for temperature at select pressure levels—pyroCb group versus large standard wildfire group, 1991-2020.

**Supplementary Table S2.** Descriptive statistics and Mann-Whitney *U*-test results for dew point at select pressure levels—pyroCb group versus large standard wildfire group, 1991-2020.


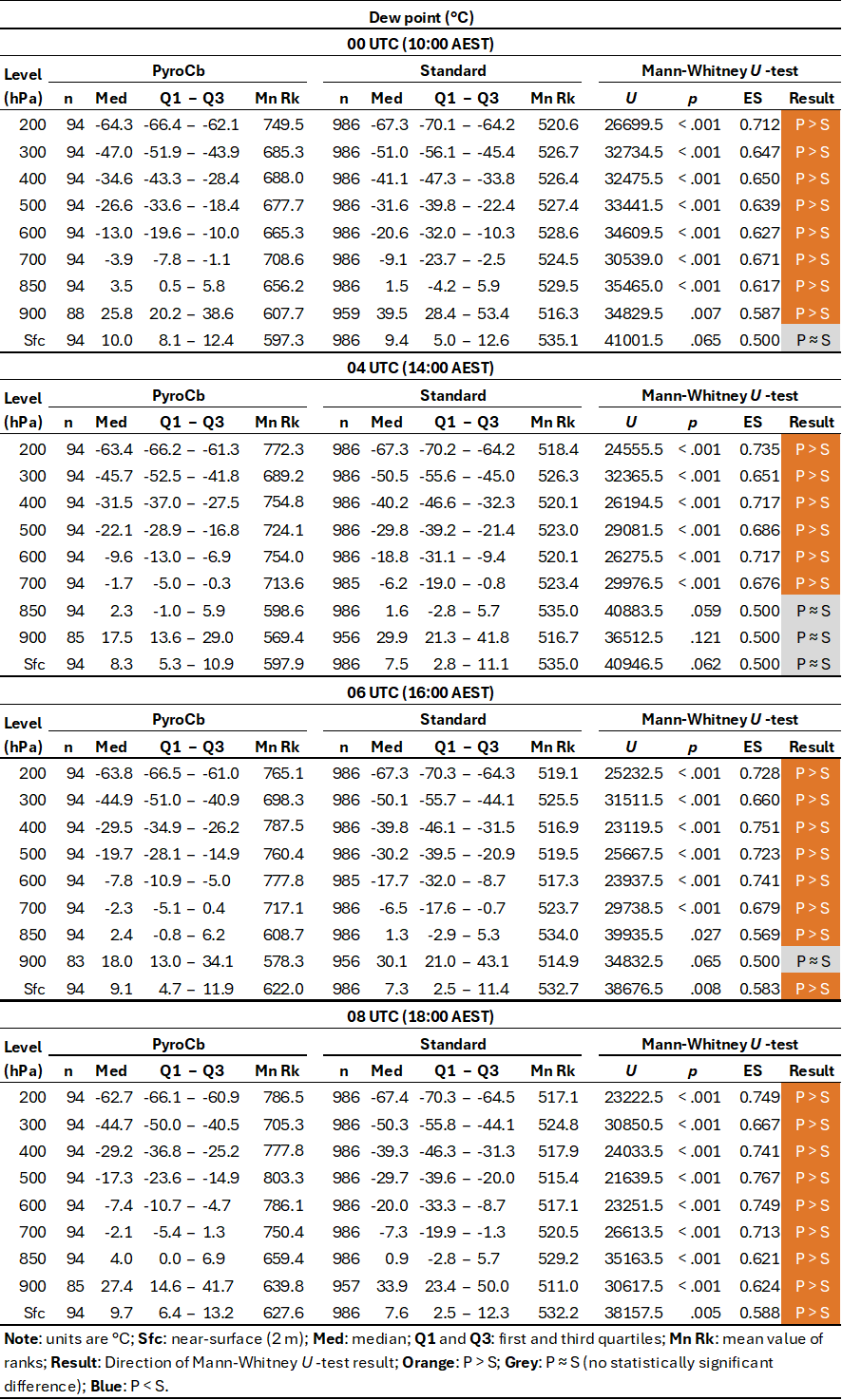


**Supplementary Table S3.** Descriptive statistics and Mann-Whitney *U*-test results for relative humidity at select pressure levels—pyroCb group versus large standard wildfire group, 1991-2020.


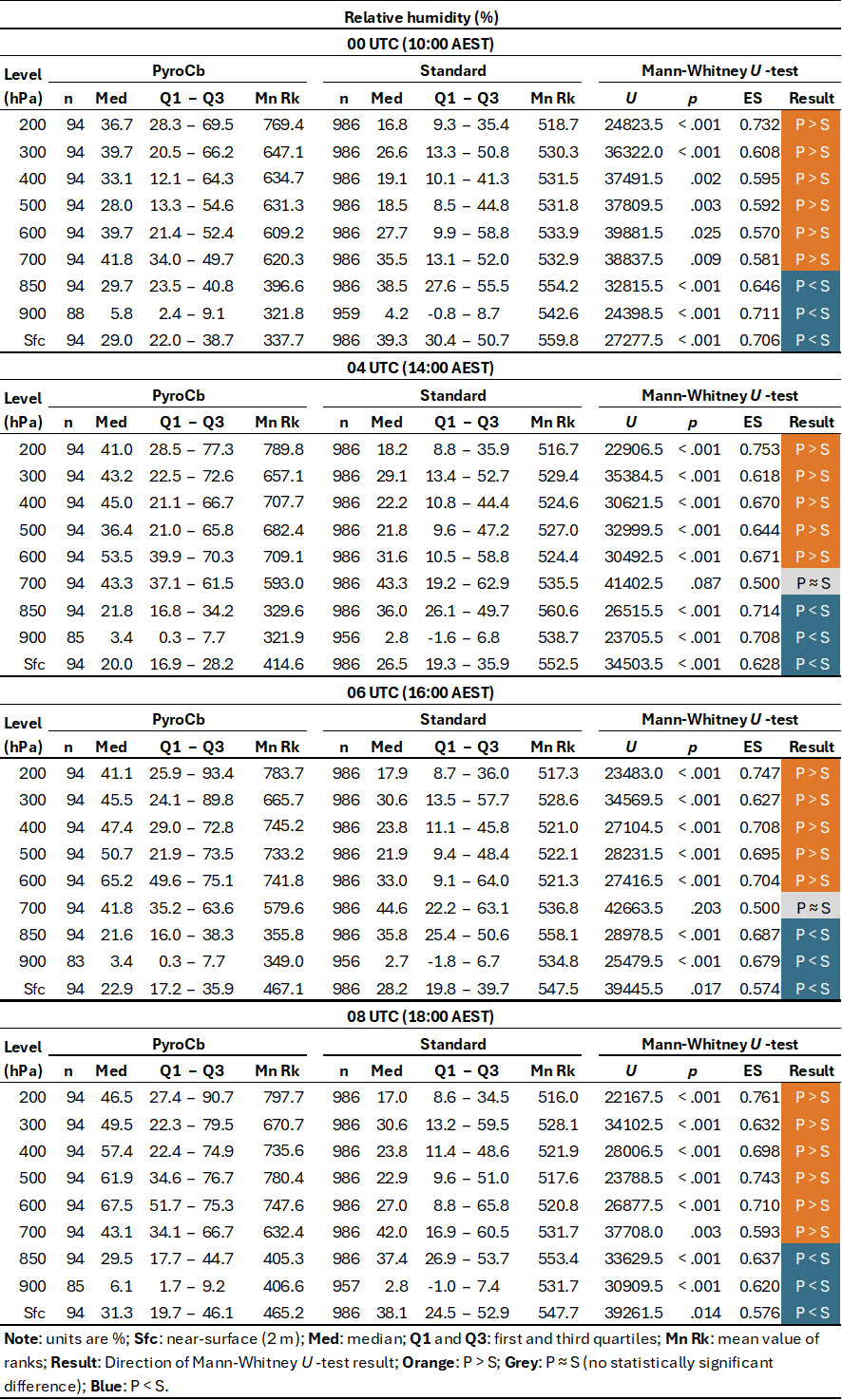


**Supplementary Table S4.** Descriptive statistics and Mann-Whitney *U*-test results for lapse rates and precipitable water vapour at select pressure levels—pyroCb group versus large standard group, 1991-2020.


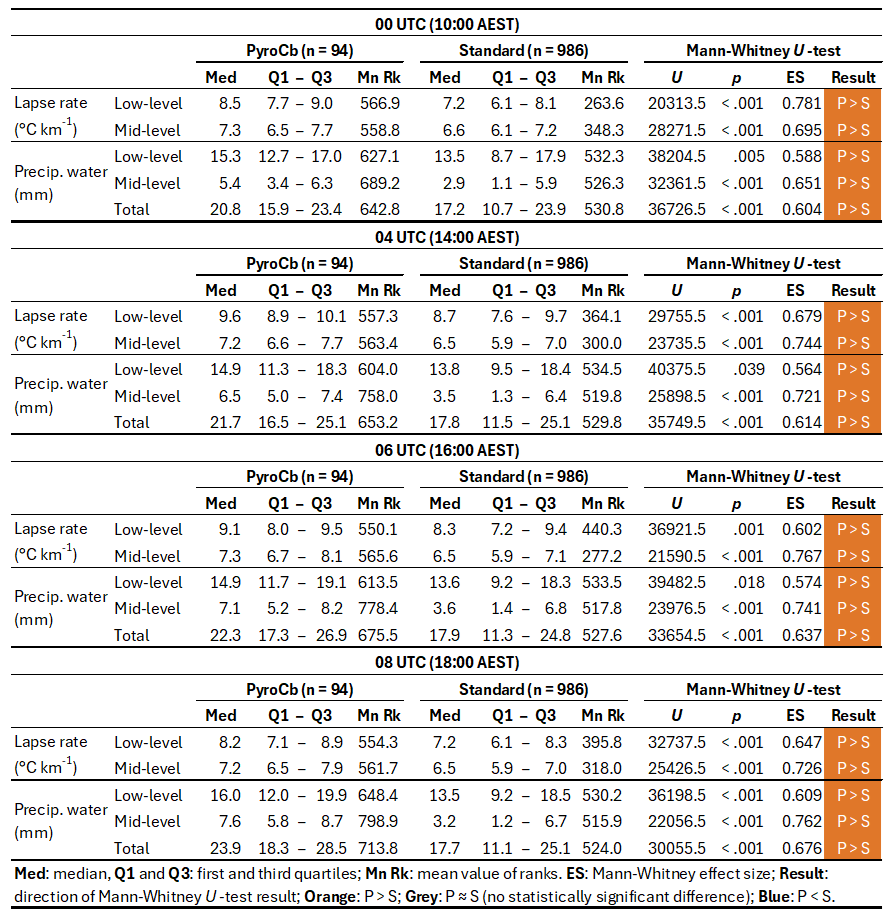


**Supplementary Table S5.** Descriptive statistics and Mann-Whitney *U*-test results for wind speed at select pressure levels—pyroCb group versus large standard wildfire group, 1991-2020.


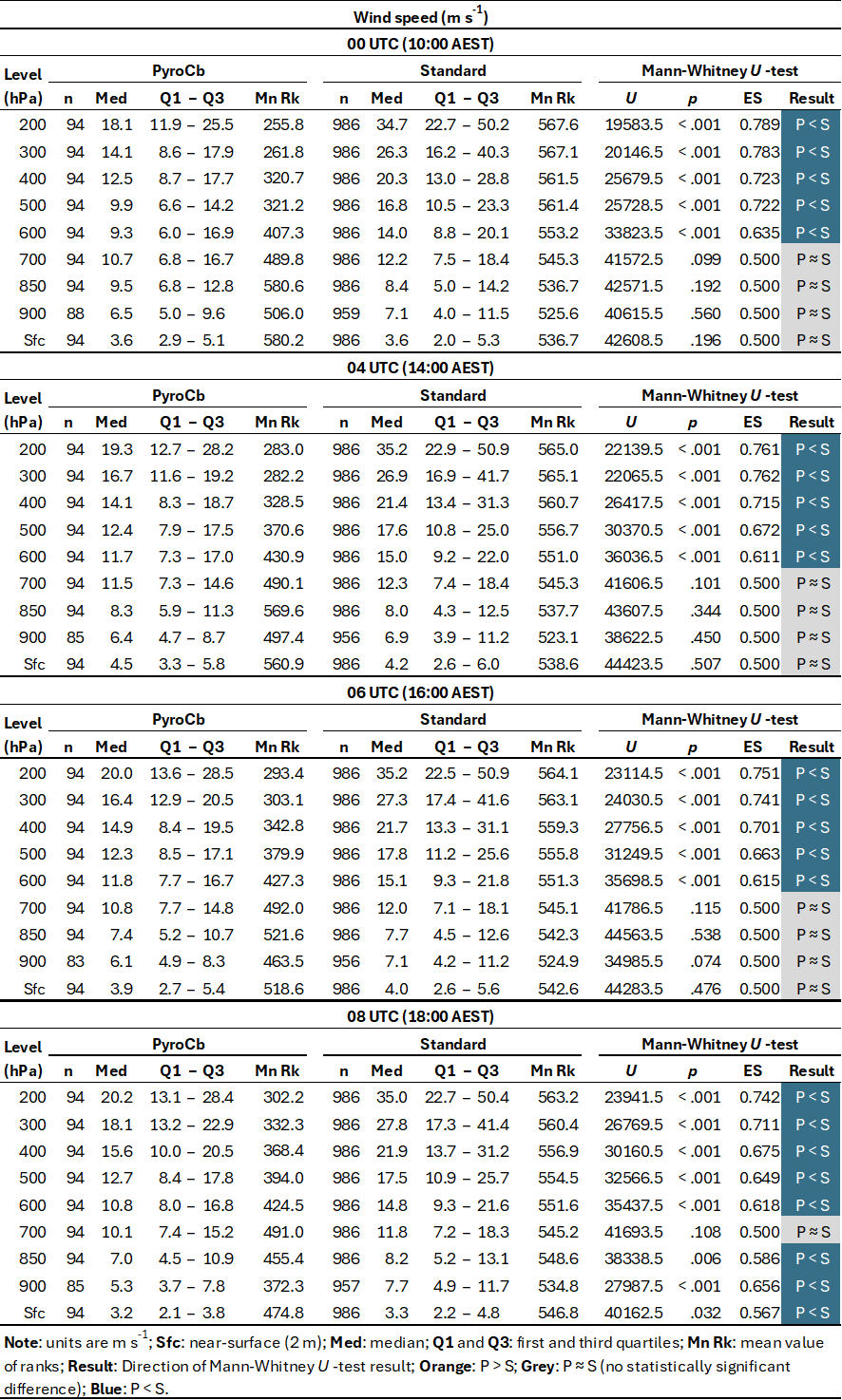

Supplement: Supplementary file 1 — Supplementary Material 1 [file 41598_2025_22530_MOESM1_ESM.docx]
